# Evaluation of knowledge and attitude concerning augmented renal clearance among physicians and clinical pharmacists in Al-Ain, UAE: A cross-sectional study

**DOI:** 10.1371/journal.pone.0310081

**Published:** 2024-09-19

**Authors:** Betoul Alshouli, Maram O. Abbas, Raniah Alsharji, Ammar Ali Saleh Jaber

**Affiliations:** 1 Department of Pharmacy Practice, Dubai Pharmacy College for Girls, Dubai Medical University, Dubai, United Arab Emirates; 2 Institute of Public Health, College of Medicine & Health Sciences, United Arab Emirates University, Al-Ain, United Arab Emirates; 3 Inpatient Pharmacy, Oncology Department, Tawam Hospital, Tawam, Abu Dhabi, United Arab Emirates; Mie University Hospital: Mie Daigaku Igakubu Fuzoku Byoin, JAPAN

## Abstract

**Background:**

Kidney function assessment is crucial in critical illness patients and is required before administering renally excreted medication, especially antibiotics and antiepileptics. Conventional clinical practice often focuses on renal impairment with low creatinine clearance (CrCl) and overlooks the augmented renal clearance (ARC), which is defined by (CrCl) more than 130 ml/min. This typical demonstration neglects individuals who experience hyperfunctioning kidneys. Among critically ill patients, the prevalence of (ARC) is approximately 20% to 65% of cases. This study aims to evaluate physicians’ and clinical pharmacists’ knowledge about ARC-associated risk factors, antibiotic regimen modification in ARC patients, and attitudes towards ARC workshops and guidelines in Al-Ain, UAE.

**Methods:**

A cross-sectional, online self-administered survey-based study was designed to achieve this study’s aim. The questionnaire was constructed on profound literature analysis, validated, and piloted. The survey was emailed to physicians and pharmacists working in two hospitals, private and governmental, and distributed through different social media platforms over three months, December 2022—February 2023.

**Results:**

Of the 92 complete responses (32 clinical pharmacists, 60 physicians), 57 (61.9%), were aware of ARC, but 72 (78%) demonstrated poor knowledge overall. Clinical pharmacists had a higher mean rank of knowledge than the physician’s group. Meanwhile, 70 (76.1%) participants were unaware of the eGFR threshold to determine ARC. There is a noticeable positive attitude toward seeking more information about antibiotic dose adjustment in ARC patients at 85 (92%) of the respondents. Remarkably, only 28 (30.4%) were directly involved with ARC patients’ treatment plans.

**Conclusion:**

In conclusion, clinical pharmacists showed better knowledge than physicians. However, overall, the participating healthcare providers lacked knowledge about ARC, so a reliable source of information regarding ARC should be utilized. Future research could explore the implementation of professional development workshops for healthcare providers and national guidelines and then assess their impact on patient outcomes.

## Introduction

Augmented renal Clearance (ARC) refers to the increase in renal elimination of circulating solutes and medicines compared to the normal range and is typically defined as creatinine clearance CrCl of more than 130 ml/min [1]. However, In respective literature, there is no agreed-upon cutoff for CrCl levels over which a patient is precisely diagnosed with ARC, nor is there a staging system for individuals with CrCl levels greater than 150 ml/min/1.73 m^2^ or even 200 ml/min/1.73 m^2^, if compared to renal impairment stages [2].

While not fully understood, this phenomenon could account for a variety of therapeutic failures for renally cleared medicines, primarily because ARC often goes unnoticed until practitioners actively monitor for its occurrence [3], adding the shortage of reliable information and guidelines regarding the dosage regimen adjustments in patients with augmented renal function, which mainly causes expedited elimination, wind-up in subtherapeutic levels and suboptimal outcomes and even treatment failure in some cases [4–7]_._

Furthermore, reports of ARC in children have been made; it is not limited to adulthood [8, 9]. Conferring to recent publications, ARC is a significant phenomenon with explicit criteria for ICU patients in adults and children [10].

The ARC state influences the bioavailability and clearance of anticoagulants, antiepileptics, β-lactams, and vancomycin [5, 11–13]. For instance, the consequence of ARC on pharmacokinetic and pharmacodynamic exposure is particularly pertinent for β-lactams antibiotics owing to their time-dependent PK/PD property and short half-life [5]. Principally, standard dosage regimens are doubtful and incompetent in keeping free antibiotic blood concentrations above the minimum inhibitory concentration in the presence of ARC, as they are time-dependent antibiotics, and the minimum required MIC differs by class. Moreover, other antibiotics, such as vancomycin, necessitate therapeutic drug monitoring to maximize clinical effectiveness and lower the risk of nephrotoxicity [14]. Vancomycin trough concentrations of 15 to 20 mg/L were traditionally thought to be an ideal surrogate for the therapeutic AUC/MIC of 400 against Methicillin-Resistant Staphylococcus Aureus (MRSA) infections with a maximum MIC of 1 mg/L and were acceptable for customizing dosing regimens [15]. The effects of ARC on vancomycin have been well documented over the past ten years because of its kidney elimination and required monitoring. Data reinforces that ARC increases vancomycin clearance and decreases the likelihood of reaching a therapeutic trough, potentially jeopardizing infected patients’ lives [12]. Hence, Ignorance of healthcare providers about ARC and its related risk factors can seriously impact the patients’ outcomes. Afterwards, this study aims to evaluate clinical pharmacists and physicians’ knowledge about risk factors associated with ARC and antibiotic regimen modification required in ARC patients, as well as their attitudes toward ARC workshops and guidelines applications in Al-Ain in UAE.

## Methods

### Study design

A survey-based, cross-sectional study was conducted to accomplish this study’s goal. Google Forms was used to create and distribute an online survey. A convenience sample has been selected. The questionnaire was distributed among physicians and clinical pharmacists in two hospitals; one is governmental, and the other is private hospital. Three different approaches were utilized to collect the responses: direct interviews with the respondents in the hospital setting, distributed via email to the hospital pharmacists and physicians, and an online link posted on different social media network platforms (Facebook, LinkedIn, and Instagram). The study was conducted over three months between December/2022 and February/2023.

### Sample size

The sample size was considered from similar knowledge and attitude studies among healthcare providers at Al-Ain City in UAE [16, 17]_._

### Inclusion and exclusion criteria

All physicians and clinical pharmacists with clinical experience who work in the following departments: ICU, Urology, Nephrology, Paediatric, Oncology, and Internal medicine were included in the study.

Healthcare providers other than physicians and clinical pharmacists from the above-listed departments were excluded.

### Data collection sheet

A questionnaire of 22 questions includes four main domains: Demographics, knowledge, and attitude. The questions have been extracted from the literature, and the changes needed to fit the purpose of our study have been made. The final questionnaire was revised and validated by three experts from the academic field and two physicians from the practical field. A pilot study was done with 20 collected responses; the Cronbach alpha score was 0.71.the questionnaire in the S1 File.

### Ethical approval

DPC Clinical Research Ethics Committee and Tawam Human Research Ethics Committee approved the protocol of this study. No personal information was asked in the survey (i.e., name or contact number). Written consent (i.e., in the form of a cover letter) was provided. A digital consent form with an agree and disagree button was presented before participation. If participants agreed to proceed, they were directed to continue the survey. Conversely, if they disagreed, the browser automatically terminated the survey.

### Data analysis

Categorical variables, such as age, gender, profession, and speciality, were represented as frequency and percentage. The Kolmogorov-Smirnov was used to assess the normality of the data. The p-value showed a non-normal distribution, so a nonparametric analysis test was used. Regarding knowledge, each question had a definitive response taken straight from the literature; this selection guaranteed the objectivity and consistency of knowledge evaluation. In addition, the survey’s design has 28 questions, enabling a comprehensive evaluation of participants’ subject-matter expertise. Each correct answer received a score of 1, while each incorrect or "unsure" answer received a zero score. The overall knowledge score was categorized into poor, moderate, and good knowledge; a score of less than 50 was categorized as poor knowledge, 50–70 as moderate knowledge and more than 70 as good knowledge. The participants’ attitude toward knowing more about ARC and implementing a proper ARC guidelines score was measured by four questions; participants gained one point for each positive response and zero for negative ones. Participants with a score of three or more are considered to have a positive attitude and, if not, a negative attitude. This study determined the classification criteria for knowledge and attitude categories based on logical considerations aligned with established methodologies in similar research contexts. While a direct reference for these criteria is unavailable, the method was developed to ensure consistency in categorizing participants’ knowledge and attitudes. The classification criteria were chosen to facilitate meaningful interpretation of the study findings.

The association between the knowledge and other demographics was studied using Pearson’s chi-square test. All data were analyzed using IBM SPSS Statistics version 29.0 for Windows® (IBM Corp., Armonk, NY, USA). A p-value ≤0.05 was considered statistically significant.

## Results

### Demographic characteristics of the study population

**Fig 1:**
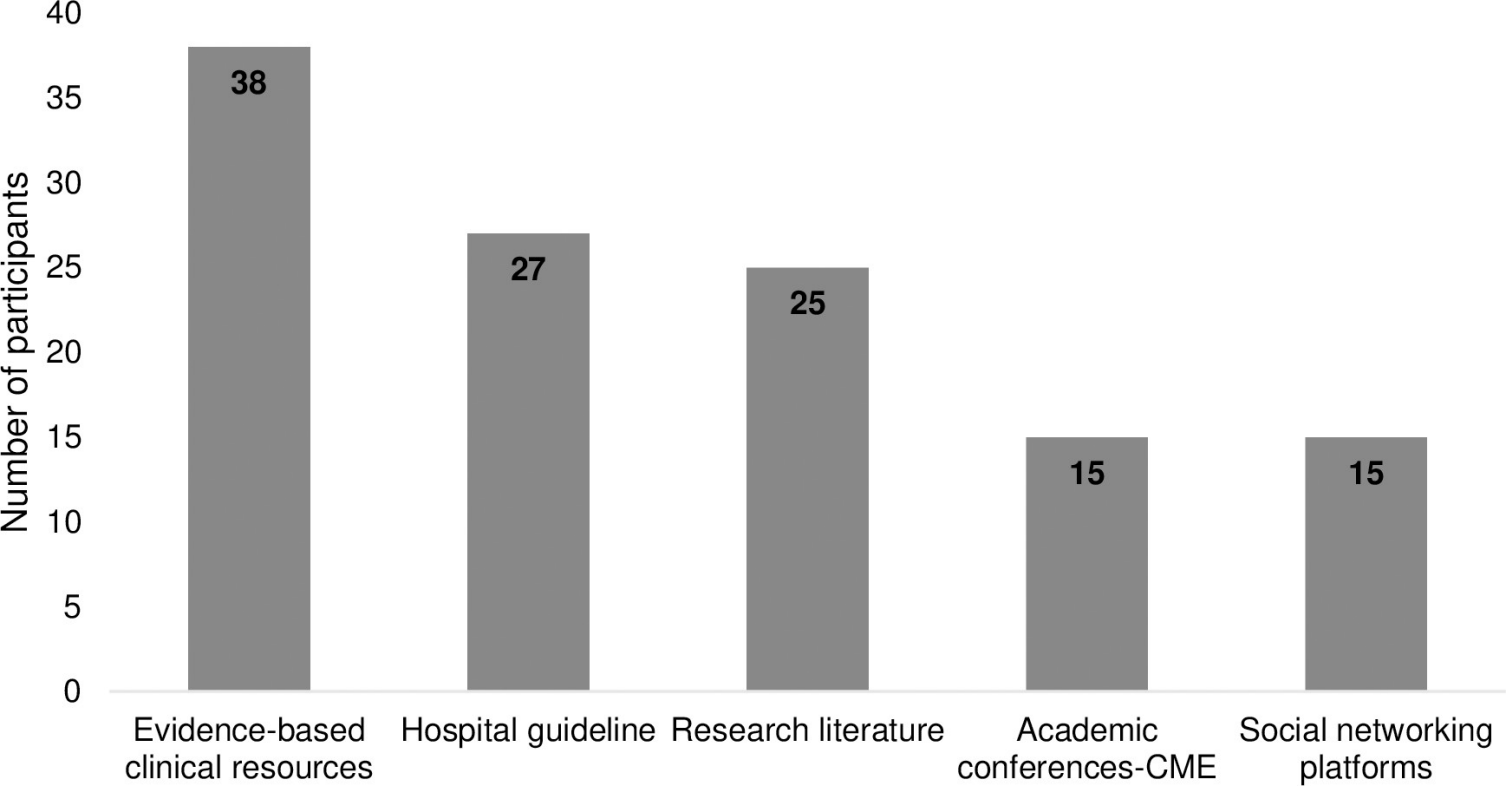
Participants’ source of information about ARC. ARC; augmented renal clearance, CME; continuing medical education.

The total number of participants involved in the study was 92, including physicians under different professional titles and clinical pharmacists. About 55% were under 35 years old. More than half of the participants, 67.4%, are female Table 1. Fig 1 illustrates the sources of information about Augmented Renal Clearance (ARC). Evidence-based clinical resources accounted for 41.3% of the sources, while hospital guidelines accounted for 29.3%.

**Table 1:** Demographic data of the participants.

Variable		N (%)
Age groups	under 35	51(55.4)
	35–44	16(17.4)
	45–54	15(16.3)
	55–64	9(9.8)
	65 and above.	1(1.1)
Gender	Male	30(32.6)
	Female	62(67.4)
Specialty	Internal Medicine	10(10.9)
	Pediatric	14(15.2)
	Oncology	10(10.9)
	ICU*	10(10.9)
	Nephrology	8(8.7)
	Urology	8(8.7)
	Clinical pharmacist	32(34.8)

ICU;Intinsive care unit.

### Physicians and clinical pharmacists’ knowledge about ARC

Approximately more than half, 57(62.0%) of the participants affirmed that they are aware of ARC, while 35(38.0%) stated that they are unaware of this condition. A scale of nine questions was used to assess the participant’s knowledge. Table 2 shows the items used to measure the knowledge score of the participants about ARC. β-lactams was the most identified antibiotic for the medications which could be affected by the ARC by 51.1%. Almost 12 participants did not recognize any risk factors, while only two could recognize all of them Table 2.

**Table 2:** Pharmacists and physicians knowledge regarding ARC.

Questions		Correct answers N(%)	Wrong answers N(%)
1. Which method is generally used to assess the renal function status of patients with ARC		35(38.0)	57(62)
2. What is the eGFR cutoff /threshold to determine ARC in a critically ill patient		22(23.9)	70(76.1)
3. Which conditions below have been identified as risk factors for ARC?	Burns	36(39.1)	56(60.9)
	Haematological	20(21.7)	72(78.3)
	malignancies	23(25.0)	69(75.0)
	Major surgery	15(16.3)	77(83.7)
	Neutrophils with fever	37(40.2)	55(59.8)
	Severe trauma	16(17.4)	76(82.6)
	Male gender	18(19.6)	74(80.4)
	Young age < 55	62(67.4)	30(32.6)
	Sepsis	25(27.2)	67(72.8)
	I am not sure	67(72.8)	25(27.2)
4. Which drug group is pharmacokinetically affected by the ARC?	β-lactams	47(51.1)	45(48.9)
	Glycopeptide	31(33.7)	61(66.3)
	Aminoglycoside	56(60.9)	36(39.1)
	Anticoagulant	15(16.3)	77(83.7)
	Antiepileptic	22(23.9)	70(76.1)
5. what are the recommended strategies to manage antibiotics in ARC patient	Use the maximum approved dosing regimen	32(34.8)	60(65.2)
	Administer doses in a prolonged or continuous infusion	28(30.4)	64(69.6)
	Therapeutic drug monitoring	63(68.5)	29(31.5)
	Switch to an alternative agent that is not largely renally eliminated	41(44.6)	51(55.4)
6. What proper modification should be made regarding the following antibiotics in the ARC setting?	Amoxicillin-Clavulanic acid	27(29.3)	65(70.7)
	Piperacillin-Tazobactam	23(39.1)	69(60.9)
	Vancomycin	4(4.3)	88(95.7)
	linezolid	3(3.3)	89(96.7)
7. What is the recommended loading dose of vancomycin in the setting of ARC for adult		30(32.6)	62(67.4)
8. What is the recommended maintenance dose of vancomycin in the setting of ARC for an adult		25(27.2)	67(72.8)
9. How would you monitor vancomycin trough level in the presence of ARC		22(23.9)	70(76.1)

ARC, Augmented renal clearance; eGFR, estimated glomerular filtration rate.

**Fig 2:**
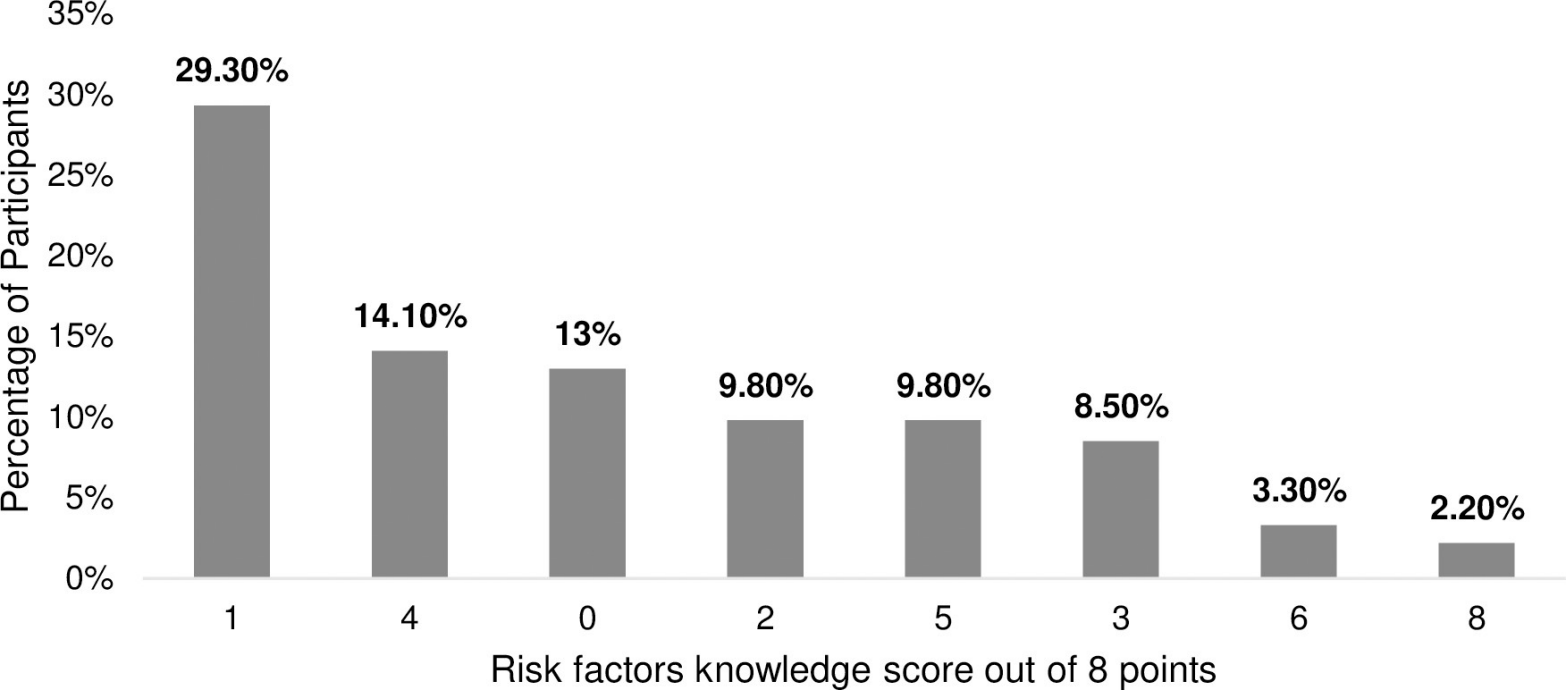
Risk factors knowledge score. ARC, augmented renal clearance.

**Fig 3:**
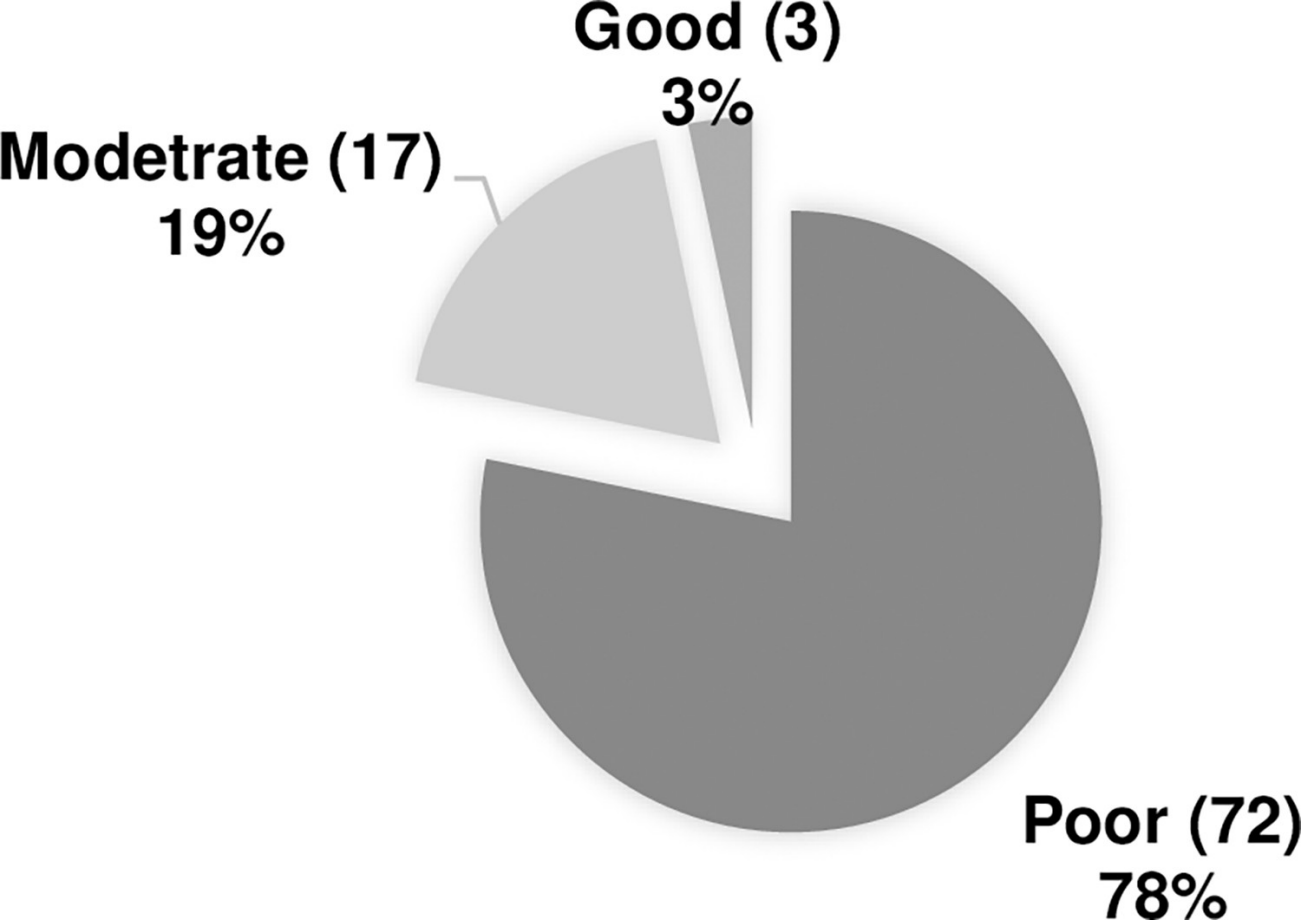
Pharmacists and physicians’ knowledge categories.

Regarding the risk factors associated with Augmented Renal Clearance (ARC), sepsis was the most recognized risk factor, with 67.4% of participants correctly identifying it, while major surgeries were the least known risk factor, recognized by only 16.3% of participants. Only 27 participants (29%) were able to identify four or more risk factors, whereas 65 participants (70.6%) did not (Fig 2). Participants were categorized into three groups based on their knowledge level, with the majority demonstrating poor knowledge. Specifically, 72 participants (78%) were categorized as having poor knowledge (Fig 3).

### Physicians and clinical pharmacists’ attitudes about ARC

**Fig 4:**
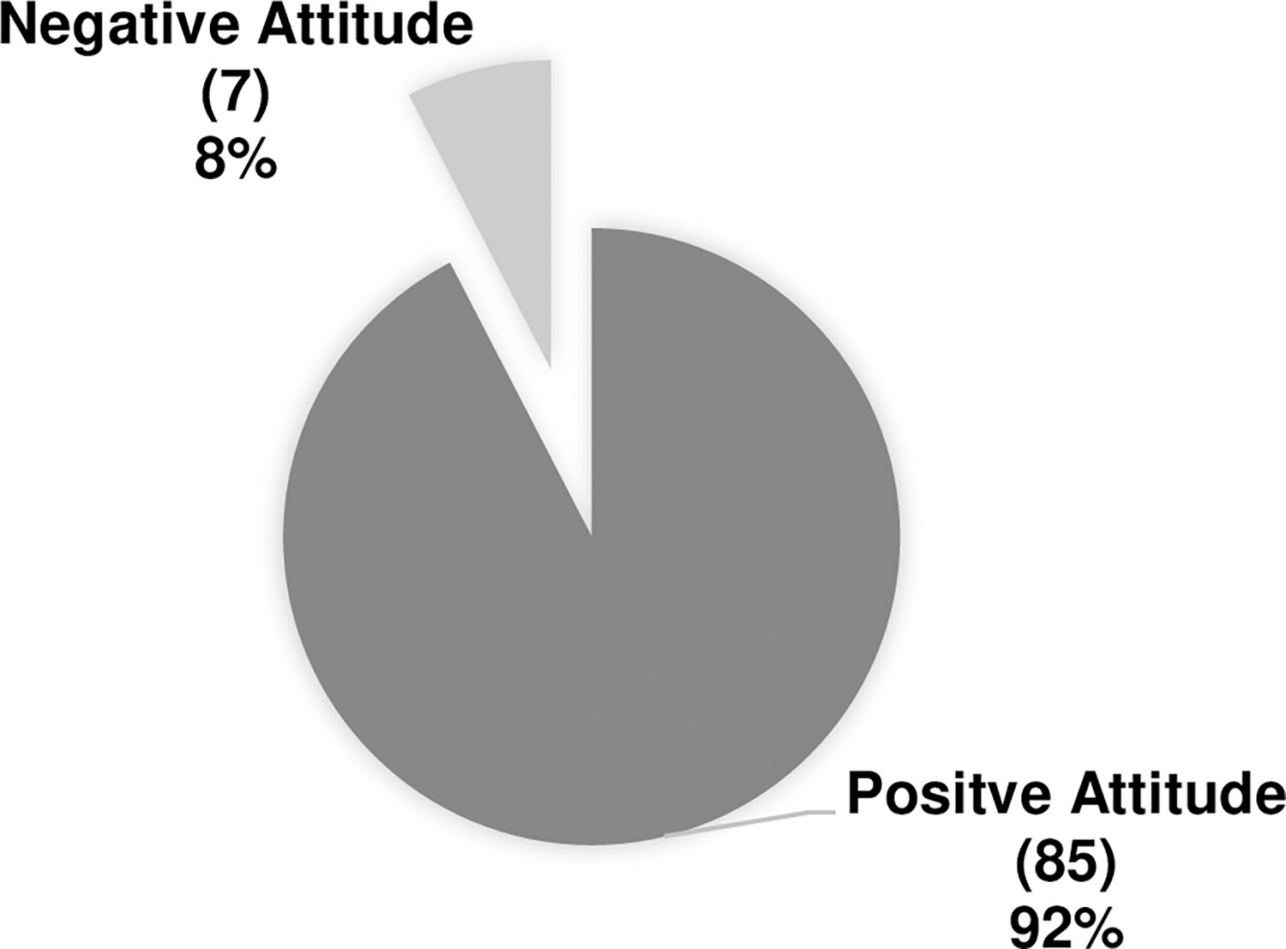
Pharmacists and physicians’ attitude categories.

Four questions were used to assess the healthcare provider’s attitude. The analysis showed that 83.7% would seek more information about antibiotic adjustment in the case of ARC. The majority, 87.0%, are interested in attending workshops about ARC. Moreover, the participants showed 96.7% willingness to apply antibiotic adjustment guidelines once established in case of ARC occurrence. The participants were categorized into two groups according to their attitudes. Most participants were positive at 85 (92%) as shown in (Fig 4). About 79 (85.9%) of respondents perceived that ARC status could affect the treatment outcomes of antibiotic therapy. Moreover, only 28 (30.4%) participants have managed patients with ARC.

### Association between participants’ demographic, knowledge, and attitude

The association between demographic factors and participants’ knowledge and attitude towards ARC have has been assessed using Chi-square analysis. Significant associations were found among two participant groups, clinical pharmacists and physicians, regarding their knowledge of ARC, with p-values of ≤0.05, suggesting a notably low level of knowledge among these medical professionals. A similar trend was observed for speciality. Additionally, significant associations were found between positive attitude and speciality, as well as particular sources of information such as research literature, hospital guidelines, and evidence-based resources, as illustrated in Table 3. Further nonparametric analysis revealed that clinical pharmacists had a significantly higher mean rank of 59.13 compared to physicians, whose mean rank was 39.77, with a significance value of less than 0.001.

**Table 3:** Association between participants’ demographic, attitude, and their knowledge.

Demographic Characteristics		Knowledge about ARC among participants (N)			Chi-Square p-value	Attitude toward ARC (N)		Chi-square P value
		Good	Moderate	Poor		Negative	Positive	
**Gender**	Female	2	13	47	0.663	6	56	0.267
	Male	1	4	25		1	29	
**Age**	Under 35 years	2	7	42	0.738	5	46	0.872
	35–44	0	3	13		1	15	
	45–54	1	4	10		1	14	
	55–64	0	3	6		0	9	
	Above 65	0	0	1		0	1	
**Profession**	Physician	3	6	51	0.010*	6	54	0.236
	Clinical pharmacist	0	11	21		1	31	
**Specialty**	Internal	1	2	7	0.028*	0	10	0.006*
	Pediatric	0	1	13		2	12	
	Oncology	0	0	10		4	6	
	Cardiology	0	0	2		0	2	
	Nephrology	0	1	7		0	8	
	Urology	0	1	7		0	8	
	ICU	2	1	5		0	8	
	Clinical pharmacist	0	11	21		1	31	
**Source of information**	Academic conferences CME	0	3	12	0.736	0	15	0.224
	Research literature	2	5	18	0.894	0	25	0.020*
	Social networks	2	2	11	0.877	2	13	0.585
	Hospital guidelines	0	4	23	0.415	1	26	0.050*
	Evidence-based resources	1	11	26	0.094	0	36	0.021*

* Significance at *P* value equal to or less than 0.05

ARC, Augmented renal clearance.

## Discussion

This study assessed physicians’ and clinical pharmacists’ knowledge and attitudes towards ARC. Findings indicate generally inadequate knowledge, consistent with a study from Saudi Arabia [18] but on a lower scale than in China [19]. Unfortunately, only a minor percentage of participants could define the ARC threshold of 130 ml/min, which is less than the Zhou et al. study with the difference that the e threshold in paediatrics is 160 ml/min [6].

Concerning the source of information, a good portion of our participants relied on evidence-based clinical resources, which contradicts the Zhou et al., 2021 study, which stated that paediatric practitioners are using social networking to gain information about ARC. However, noticeably, participants under 35 depended on the social networking platform to get information. The same result was shown in a similar study by [19].

Moreover, As defined in the literature, those risk factors can increase the probability of being vulnerable to ARC: burns, young age <55, male gender, febrile neutropenia, haematological disorder, malignancies, trauma, and sepsis [5, 20–24]. Only 2.2% could define all risk factors aligned, while only 0.7% of the Almulhim Batool et al. study participants achieved it [18]. Comparative analysis indicates that participants in this study had lower awareness of risk factors for ARC than the 70% observed by Zhou et al. [19]. While the study identified all significant risk factors, the only one widely recognized among participants was being under 55 years of age. Although severe trauma is a well-established risk factor for ARC, it was acknowledged by only a minority of participants [25].

A remarkable finding is that the clinical pharmacist achieved a higher knowledge score than physicians with a significant value, which is the opposite of the findings of the Almulhim et al. 2020 study [8]. Their study was among inpatient pharmacists (with clinical experience) and concluded that pharmacists had limited knowledge about ARC.

Furthermore, around 38% of participants chose the accurate method to evaluate ARC, which is urine collection, and it is the only precise method to measure CRCL in ARC patients [26].

The previous research revealed that β-lactams, aminoglycosides, glycopeptides, anticoagulants, and antiepileptics are among the medications that are impacted by the ARC [7, 8, 11, 19, 27–29]. This effect can cause subtherapeutic levels, which could lead to treatment failure [30]. Half the participants agreed that ARC affects β-lactams antibiotics glycopeptides, but less than a quarter identified anticoagulant and antiepileptic.

Aminoglycosides are an additional medication to be mentioned because of their reputation for renal clearance [7]. Nevertheless, the literature found that ARC incidence does not affect the pharmacokinetics-dynamics of aminoglycoside, although 36 participants chose it as an affected antibiotic [7].

It is imperative to optimize the dosing regimen for patients who require antibiotics and are susceptible to ARC. Two primary approaches exist for managing such patients since enhanced clearance results in subtherapeutic concentrations, which can endanger the patient’s life. The first is to increase the dosing regimen to the upper known limit as much as possible. The other approach is to change the administration time into extended or continuous infusions to benefit from time-dependent antibiotics [20]. Less than half of the participants could recognize the proper methods of dealing with ARC patients when it comes to managing antibiotic dosing; these numbers oppose Almulhim et al., 2020 study as they showed the appropriate dose and frequency of administration of piperacillin-tazobactam in ARC patients were chosen by the majority of respondents (60.4%) [18].

More profound questions were used to clarify the knowledge concerning β-lactams and vancomycin for their high prescription rate, and they were considered among the most prescribed antibiotics in the ICU units [31]. As approved in the literature, the proper action is to extend the infusion. Only 6% recognize it, but increasing the dose is another successful approach to dealing with ARC patients [32].

Surprisingly, about 27% choose to decrease the dose in the case of ARC, which is the opposite and wrong practice [25]. Only three respondents chose the appropriate modification for Linezolid to extend the infusion time; as Barrasa et al. study stated, "augmented renal clearance (ARC) increases linezolid clearance and leads to a high risk of underexposure with the standard dose. Continuous infusion increases the PTA (PK/PD target attainment) [33].

As well known, vancomycin is a game changer in critically ill patients as a final resort to treat resistant bacteria, and practitioners must be aware of its loading dose, maintenance dosing, and monitoring [34–36]. Unfortunately, only a quarter of the participant practitioners knew the proper management of vancomycin in ARC patients, which complies with the Zhou et al., 2021 study [19]. These results showed that current practitioners do not recognize the new guideline to monitor vancomycin levels by AUC/MIC [14]. Our study had some limitations. Due to geographical limitations, we could only involve one city in the UAE; this resulted in a small sample size, making it difficult to generalize our findings to the GCC region. The study was conducted over a relatively short period (three months), which may not capture potential variations in knowledge and practices over more extended periods.

## Conclusion and recommendation

We concluded that physicians and clinical pharmacists know little about ARC. Half of the participants could not answer the knowledge-related questions correctly. The difference in knowledge score between clinical pharmacists and physicians is significant, indicating that the clinical pharmacy is more knowledgeable. We recommend that policymakers give special attention to educational programs to fill the knowledge gap in ARC management among medical professionals. Conducting more webinars and continuing medical education (CME) programs could effectively educate healthcare providers about ARC, especially since they are willing to apply established guidelines and are open to attending related conferences. These initiatives should include comprehensive training programs and guidelines covering ARC recognition, risk factors, and optimal antibiotic dosing strategies to prevent treatment failure in this vulnerable population.

## Supporting information

S1 Data SPSS miniset of raw data coded. This file contains the raw, coded data in SPSS format used for statistical analysis in the study.
(SAV)

S1 File Survey instrument used in the study. This file includes the complete survey questionnaire administered to participants, detailing the questions and response options used to assess knowledge, attitudes, and behaviors related to the study topic.
(DOCX)

## References

[1] Mahmoud SH, Shen C. Augmented renal clearance in critical illness: An important consideration in drug dosing. Pharmaceutics. 2017;9(3):36. doi: 10.3390/pharmaceutics9030036 28926966 PMC5620577

[2] Cook AM, Hatton-Kolpek J. Augmented Renal Clearance. Vol. 39, Pharmacotherapy. Pharmacotherapy Publications Inc.; 2019. p. 346–54. doi: 10.1002/phar.2231 30723936

[3] Mikami R, Hayakawa M, Imai S, Sugawara M, Takekuma Y. Onset timing and duration of augmented renal clearance in a mixed intensive care unit. J Intensive Care. 2023 Dec 1;11(1). 10.1186/s40560-023-00660-9 PMC10035487 36959656

[4] Baptista JP, Udy AA, Sousa E, Pimentel J, Wang L, Roberts JA, et al. A comparison of estimates of glomerular filtration in critically ill patients with augmented renal clearance. Crit Care. 2011;15(3). doi: 10.1186/cc10262 21651804 PMC3219011

[5] Udy AA, Roberts JA, Boots RJ, Paterson DL, Lipman J. Augmented renal clearance: Implications for antibacterial dosing in the critically ill. Clin Pharmacokinet. 2010;49(1):1–16. doi: 10.2165/11318140-000000000-00000 20000886

[6] Van Der Heggen T, Dhont E, Peperstraete H, Delanghe JR, Vande Walle J, De Paepe P, et al. Augmented renal clearance: a common condition in critically ill children. Pediatr Nephrol. 2019 Jun 1;34(6):1099–106. doi: 10.1007/s00467-019-04205-x 30778827

[7] Avedissian SN, Rhodes NJ, Kim Y, Bradley J, Valdez JL, Le J. Augmented renal clearance of aminoglycosides using population-based pharmacokinetic modelling with Bayesian estimation in the pediatric ICU. J Antimicrob Chemother. 2020;75(1):162–169. doi: 10.1093/jac/dkz398 31648297

[8] Avedissian SN, Bradley E, Zhang D, Bradley JS, Nazer LH, Tran TM, et al. Augmented Renal Clearance Using Population-Based Pharmacokinetic Modeling in Critically Ill Pediatric Patients*. Pediatr Crit Care Med. 2017 Sep 1;18(9):e388–94. 28640009 10.1097/PCC.0000000000001228

[9] Dhont E, Van Der Heggen T, De Jaeger A, Vande Walle J, De Paepe P, De Cock PA. Augmented renal clearance in pediatric intensive care: Are we undertreating our sickest patients? Pediatr Nephrol. 2020;35(1):25–39. doi: 10.1007/s00467-018-4120-2 30374606

[10] Luo Y, Wang Y, Ma Y, Wang P, Zhong J, Chu Y. Augmented Renal Clearance: What Have We Known and What Will We Do? Vol. 12, Frontiers in Pharmacology. Frontiers Media S.A.; 2021. 10.3389/fphar.2021.723731 PMC8593401 34795579

[11] bdel El Naeem HEM, Abdelhamid MHE, Atteya DAM. Impact of augmented renal clearance on enoxaparin therapy in critically ill patients. Egypt J Anaesth. 2017;33(1):113–117. doi: 10.1016/j.egja.2016.12.007

[12] Chu Y, Luo Y, Qu L, Zhao C, Jiang M. Application of vancomycin in patients with varying renal function, especially those with augmented renal clearance. Pharm Biol. 2016;54(12):2802–2806. doi: 10.1080/13880209.2016.1183684 27251880

[13] Béranger A, Benaboud S, Urien S, Moulin F, Bille E, Lesage F, et al. Piperacillin population pharmacokinetics and dosing regimen optimization in critically ill children with normal and augmented renal clearance. Clin Pharmacokinet. 2019;58(2):223–233. doi: 10.1007/s40262-018-0682-1 29862466

[14] Alosaimy S, Murray KP, Zasowski EJ, Morrisette T, Lagnf AM, Lodise TP, et al. Vancomycin Area Under the Curve to Predict Timely Clinical Response in the Treatment of Methicillin-resistant Staphylococcus aureus Complicated Skin and Soft Tissue Infections. Clin Infect Dis. 2020 Jul 27; 10.1093/cid/ciaa1039 PMC8662764 32716506

[15] Rybak M, Lomaestro B, Rotschafer JC, Moellering R, Craig W, Billeter M, et al. Therapeutic monitoring of vancomycin in adult patients: A consensus review of the American Society of Health-System Pharmacists, the Infectious Diseases Society of America, and the Society of Infectious Diseases Pharmacists. Vol. 66, American Journal of Health-System Pharmacy. American Society of Health-Systems Pharmacy; 2009. p. 82–98. 10.2146/ajhp080434 19106348

[16] AlMansoori LS, AlKatheeri MS, AlHallami AA, AlMarzooqi MY, Al-Tatari M, L-Tatari HA. Physicians’ knowledge, attitude, and practices toward HPV disease and vaccination in Al Ain city, UAE. Int J Contemp Res Rev. 2019 Jun 17;10(06):20741–50.

[17] Ortashi O, Shallal M, Osman N, Raheel H. Knowledge, attitude, and practice of school nurses in the United Arab Emirates about HPV infection and vaccine. Asian Pac J Cancer Prev. 2012;13(12):6481–6484. Available from: https://pubmed.ncbi.nlm.nih.gov/23464478/ doi: 10.7314/apjcp.2012.13.12.6481 23464478

[18] Almulhim BA, Al-Dahneen YS, Alsowaida AS. Pharmacists’ knowledge about the impact of augmented renal clearance on antimicrobial dosing in critically ill patients: A cross-sectional study. Infect Dis Ther. 2020;9(4):827–841. doi: 10.1007/s40121-020-00310-9 32594458 PMC7452990

[19] Zhou R, Fang Y, Wang C, Zhou S. Knowledge, attitudes, and practices related to augmented renal clearance among pediatricians in China: A cross-sectional study. Med (United States). 2021 Aug 13;100(32):E26889. 10.1097/MD.0000000000026889 PMC8360415 34397910

[20] Lv C Le, Lu JJ, Chen M Zhang R, Li QC, Chen YY, et al. Vancomycin population pharmacokinetics and dosing recommendations in haematologic malignancy with augmented renal clearance children. J Clin Pharm Ther. 2020;45(6):1278–87. doi: 10.1111/jcpt.13206 32557716

[21] Baptista JP, Martins PJ, Marques M, Pimentel JM. Prevalence and Risk Factors for Augmented Renal Clearance in a Population of Critically Ill Patients. J Intensive Care Med. 2020;35(10):1044–52. doi: 10.1177/0885066618809688 30373438

[22] Cojutti PG, Lazzarotto D, Candoni A, Dubbini MV, Zannier ME, Fanin R, et al. Real-Time TDM-based optimization of continuous-infusion meropenem for improving treatment outcome of febrile neutropenia in oncohaematological patients: Results from a prospective, monocentric, interventional study. J Antimicrob Chemother. 2020;75(10):3029–37. doi: 10.1093/jac/dkaa267 32681168 PMC7678894

[23] Mulder MB, Eidelson SA, Sussman MS, Schulman CI, Lineen EB, Iyenger RS, et al. Risk factors and clinical outcomes associated with augmented renal clearance in trauma patients. J Surg Res. 2019;244:477–483. doi: 10.1016/j.jss.2019.06.087 31330291

[24] Loirat P, Rohan J, Baillet A, Beaufils F, David R, Chapman A. Increased Glomerular Filtration Rate in Patients with Major Burns and Its Effect on the Pharmacokinetics of Tobramycin. http://dx.doi.org/101056/NEJM197810262991703 [Internet]. 2010 Jan 13 [cited 2023 Nov 12];299(17):915–9. Available from: https://www.nejm.org/doi/full/10.1056/NEJM197810262991703 10.1056/NEJM197810262991703 692596

[25] Sime FB, Udy AA, Roberts JA. Augmented renal clearance in critically ill patients: Etiology, definition and implications for beta-lactam dose optimization. Curr Opin Pharmacol [Internet]. 2015;24:1–6. Available from: doi: 10.1016/j.coph.2015.06.002 26119486

[26] Carlier M, Dumoulin A, Janssen A, Picavet S, Vanthuyne S, Van Eynde R, et al. Comparison of different equations to assess glomerular filtration in critically ill patients. Intensive Care Med. 2015;41(3):427–435. doi: 10.1007/s00134-014-3641-9 25619485

[27] Carrié C, Chadefaux G, Sauvage N, De Courson H, Petit L, Nouette-gaulain K, et al. Increased β-Lactams dosing regimens improve clinical outcome in critically ill patients with augmented renal clearance treate. 2019;1–9. 10.1186/s13054-019-2621-4 PMC6881978 31775840

[28] Udy AA, Varghese JM, Altukroni M, Briscoe S, McWhinney BC, Ungerer JP, et al. Subtherapeutic initial β-lactam concentrations in select critically Ill patients: Association between augmented renal clearance and low trough drug concentrations. Chest. 2012;142(1):30–9. 22194591 10.1378/chest.11-1671

[29] Huttner A, Von Dach E, Renzoni A, Huttner BD, Affaticati M, Pagani L, et al. Augmented renal clearance, low β-lactam concentrations and clinical outcomes in the critically ill: an observational prospective cohort study. Int J Antimicrob Agents. 2015;45(4):385–92. Available from: 10.1016/j.ijantimicag.2014.12.017. 25656151

[30] Udy AA, Dulhunty JM, Roberts JA, Davis JS, Webb SA, Bellomo R, et al. Association between augmented renal clearance and clinical outcomes in patients receiving β-lactam antibiotics: A multicentre prospective observational study. J Antimicrob Chemother. 2015;70(3):914–920. doi: 10.1093/jac/dku453 25480492

[31] Perl TM. The threat of vancomycin resistance. Am J Med. 1999;106(5 A). doi: 10.1016/s0002-9343(98)00354-4 10348061

[32] Carlier M, Noë M, De Waele JJ, Stove V, Verstraete AG, Lipman J, et al. Population pharmacokinetics and dosing simulations of amoxicillin/clavulanic acid in critically ill patients. J Antimicrob Chemother. 2013;68(11):2600–8. F. doi: 10.1093/jac/dkt240 23800901

[33] Barrasa H, Soraluce A, Usón E, Sainz J, Martín A, Sánchez-Izquierdo JÁ, et al. Impact of augmented renal clearance on the pharmacokinetics of Linezolid: Advantages of continuous infusion from a pharmacokinetic/pharmacodynamic perspective. Int J Infect Dis. 2020 Apr 1;93:329–38. doi: 10.1016/j.ijid.2020.02.044 32112965

[34] Pea F, Furlanut M, Negri C, Pavan F, Crapis M, Cristini F, et al. Prospectively validated dosing nomograms for maximizing the pharmacodynamics of vancomycin administered by continuous infusion in critically ill patients. Antimicrob Agents Chemother. 2009;53(5):1863–7. doi: 10.1128/AAC.01149-08 19223642 PMC2681515

[35] Jeurissen A, Sluyts I, Rutsaert R. A higher dose of vancomycin in continuous infusion is needed in critically ill patients. Int J Antimicrob Agents. 2011;37(1):75–7. Available from: doi: 10.1016/j.ijantimicag.2010.09.004 21074374

[36] Kim AJ, Lee JY, Choi SA, Shin WG. Comparison of the pharmacokinetics of vancomycin in neurosurgical and non-neurosurgical patients. Int J Antimicrob Agents. 2016;48(4):381–7. doi: 10.1016/j.ijantimicag.2016.06.022 27546217

