# Supplementary material for: Evaluation of knowledge and attitude concerning augmented renal clearance among physicians and clinical pharmacists in Al-Ain, UAE: A cross-sectional study
Source: PLoS One. 2024 Sep 19;19(9):e0310081. doi: 10.1371/journal.pone.0310081 (PMC11412532; doi:10.1371/journal.pone.0310081)
Supplement: S1 File — This file includes the complete survey questionnaire administered to participants, detailing the questions and response options used to assess knowledge, attitudes, and behaviors related to the study topic. (DOCX) [file pone.0310081.s002.docx]

**Evaluation of Knowledge and attitude concerning Augmented Renal Clearance among physicians and clinical pharmacists in Al-Ain, UAE**

**Informed Consent**

You are invited to participate in a research study aims to explore the status of knowledge, attitude and practice about augmented renal clearance among physicians. Participation in this study is voluntary. By completing this survey, you are consenting to participate in this study.

The information you will share with us if you participate in this study will be kept completely confidential to the full extent of the law. No personal questions will be asked about. Data will be accessed for statistical analysis only.

Thank you for your assistance.

**DEMOGRAPHIC** **ASSESSMENT**

1. Do you work in the UAE's health sector? *Mark only one oval.*

- Yes
- No

1. What is your gender? *Mark only one oval.*

- Male
- Female

1. What is your age? *Mark only one oval.*

- under 35
- 35- 44
- 45-54
- 55-64
- 65 and above.

1. What is your professional title? *Mark only one oval.*

- Resident Fellow
- Attending physician
- Associate chief physician
- Chief physician
- Specialist
- Consultant
- Clinical pharmacist

1. What is your specialty? * *Mark only one oval.*

- General practitioner
- Emergency Medicine
- General Surgery
- Internal Medicine
- Obstetrics and Gynecology
- Pediatric
- Neurology
- Oncology
- Cardiology
- Nephrology
- Neurosurgery
- Urology
- Endocrinology
- Clinical pharmacist
- Other; **please specify**

1. Are you aware what is augmented renal clearance-ARC? **Mark only one oval.*

- Yes
- No

1. What is your augmented renal clearance-ARC Information's source? *Check all that apply.*

- Academic conferences-CME
- Research literature
- Social networking platforms
- Hospital guideline
- Evidence based clinical resources
- Other, please specify

**Knowledge Assessment Section**

1. Which of the conditions listed below have been identified as risk factors for augmented renal clearance-ARC?

Check all that apply.

- Burns
- Hematological malignancies
- Lower disease severity
- Major surgery
- Neutrophils with fever
- Severe trauma
- Male gender
- Young age < 55
- Sepsis
- Unsure

1. Which method is generally used to assess the renal function status of patient with augmented renal clearance-ARC? *Mark only one oval.*

- Clearance of iohexol
- CysC
- Schwartz formula
- Urine collection
- I don't know

1. What is the eGFR cutoff /threshold to determine augmented renal clearance-ARC in* critically ill patient? *Mark only one oval.*

- ≥110mL/min/1.73m2
- ≥130mL/min/1.73m2
- ≥160mL/min/1.73m2
- Age-dependent ARC thresholds
- Unsure

1. Which drug group pharmacokinetic affected by the augmented renal clearance ARC? * Select All Applicable *Check all that apply.*

- B-lactam
- Glycopeptide
- Aminoglycoside
- Anticoagulant
- Antiepileptic
- Other; please specify

1. what are the recommended strategies to manage antibiotics in Augmented renal clearance patient? *Check all that apply.*
   - Use maximum approved dosing regimen
   - Administer doses in a prolonged or continuous infusion
   - Therapeutic drug monitoring
   - Switch to an alternative agent that is not largely renally eliminated
2. What is the proper modification should be done regarding each of the following antibiotics in setting of Augmented renal clearance-ARC?

***Mark only one oval per row.***

Increase

the

dose

Reduce

the

dose

Increase the

frequency of

administration

Reduce the

frequency of

administration

Extend

the

infusion

time

Unsure

Amoxicillin-

clavulanic

acid

Piperacillin-

tazobactam

Vancomycin

Linezolid

1. What is the recommended loading dose of vancomycin in the setting of augmented renal clearance for adult? *Mark only one oval.*

- 15-20 mg/kg
- 25 to 30 mg/kg
- 35 mg/kg
- Unsure

1. What is the recommended maintenance dose of vancomycin in the setting of augmented renal clearance for adult? *Mark only one oval.*

- 15 to 20 mg/kg every 8–12 h
- 35mg to 40mg/kg every 12hrs
- 15 mg to 20mg/kg every 6 hours
- Unsure

1. How would you monitor vancomycin trough level in the presence of Augmented renal * clearance-ARC?

N.B: AUC24 is the new recommended methodology of vancomycin concentration monitoring.

*Mark only one oval.*

- AUC/MIC > 400 mcg*hr/mL
- AUC/MIC of 400 to 600 mcg*hr/mL
- AUC/MIC < 400 mcg*hr/mL

**ATTITUDE ASSESSMENT**

1. Does augmented renal clearance-ARC status affect the treatment outcome of antibiotic therapy? Mark only one oval.

Yes

No

1. Are you seeking for more information on antibiotic dose adjustment in setting of augmented renal clearance-ARC? *Mark only one oval.*

- Yes
- No

1. Are you interested in attending a workshop\webinar about augmented renal clearance- (ARC)? *Mark only one oval.*

- Yes
- No

1. If specific guidelines were established for augmented renal clearance-ARC medication dosing regimens, would you be interested in applying them? *Mark only one oval.*

- YES
- No

1. Are you willing to adjust the antibiotic treatment regimen according to the guidelines in cases of augmented renal clearance-ARC? *Mark only one oval.*

- Yes
- No

**Practice Assessment**

1. Have you ever managed a patient with augmented renal clearance-ARC? * *Mark only one oval.*

- Yes

No

Thank you for your time.
